# Histological and Biological Response to Different Types of Biomaterials: A Narrative Single Research Center Experience over Three Decades

**DOI:** 10.3390/ijerph19137942

**Published:** 2022-06-28

**Authors:** Margherita Tumedei, Eitan Mijiritsky, Carlos Fernando Mourão, Adriano Piattelli, Marco Degidi, Carlo Mangano, Giovanna Iezzi

**Affiliations:** 1 Department of Biomedical, Surgical and Dental Sciences, University of Milano, 20122 Milano, Italy; margytumedei@yahoo.it; 2 Retrieval Bank of the Laboratory for Undemineralized Hard Tissue Histology, University “G. D’Annunzio” of Chieti-Pescara, 66100 Chieti, Italy; gio.iezzi@unich.it; 3 Department of Otolaryngology, Head and Neck Surgery and Maxillofacial Surgery, Tel-Aviv Sourasky Medical Center, Sackler School of Medicine, Tel-Aviv University, Tel Aviv 64239, Israel; mijiritsky@bezeqint.net; 4 Goldschleger School of Dental Medicine, Sackler School of Medicine, Tel-Aviv University, Tel Aviv 39040, Israel; 5 Clinical Research Unit of the Antonio Pedro Hospital, Fluminense Federal University, Niteroi 24033-900, Brazil; mouraoufrj@yahoo.com.br; 6 Faculty of Health Science, Catholic University of San Antonio de Murcia (UCAM), 30107 Murcia, Spain; 7 Fondazione Villaserena per la Ricerca, 65013 Città Sant’Angelo, Italy; 8 Casa di Cura Villa Serena del Dott. L. Petruzzi, 65013 Città Sant’Angelo, Italy; 9 School of Dentistry, Saint Camillus International University for Health Sciences (UniCamillus), Via di Sant’Alessandro, 8, 00131 Rome, Italy; 10 Independent Researcher, 40100 Bologna, Italy; info@degidi.it; 11 Independent Researcher, Gravedona, 22100 Como, Italy; camangan@gmail.com; 12 Department of Medical, Oral and Biotechnological Sciences, University “G. D’Annunzio” of Chieti-Pescara, 66100 Chieti, Italy

**Keywords:** bone substitutes, grafts, scaffolds, tissue engineering

## Abstract

Background: In more than three decades of work of the Retrieval Bank of the Laboratory for Undemineralized Hard Tissue Histology of the University of Chieti-Pescara in Italy, many types of biomaterials were received and evaluated. The present retrospective review aimed to evaluate the histological and biological aspects of the evaluated bone substitute biomaterials. Methods: In the present study, the authors prepared a retrospective analysis after the screening of some databases (PubMed, Scopus, and EMBASE) to find papers published from the Retrieval Bank of the Laboratory for Undermineralized Hard Tissue Histology of the University of Chieti-Pescara analyzing only the papers dealing with bone substitute biomaterials and scaffolds, in the form of granules and block grafts, for bone regeneration procedures. Results: Fifty-two articles were found, including in vitro, in vivo, and clinical studies of different biomaterials. These articles were evaluated and organized in tables for a better understanding. Conclusions: Over three decades of studies have made it possible to assess the quality of many bone substitute biomaterials, helping to improve the physicochemical and biological properties of the biomaterials used in daily clinical practice.

## 1. Introduction

Studies related to bone substitute biomaterials derive from a necessity for biomaterials to help new bone formation, making it possible to reconstruct bone defects, while maintaining the biological and mechanical functions of the restored tissue [1,2,3]. Research on all biomaterials is necessary to ensure optimal results and the patients’ safety [4,5,6]. Over more than three decades, many specimens of several types of biomaterials have been received and treated to obtain thin ground sections in the Retrieval Bank of the Laboratory for Undermineralized Hard Tissue Histology of the University of Chieti-Pescara in Italy. Histological and histo-morphometric analysis of the bone response with different grafts in different clinical situations associated to the in vitro response on cell cultures are certainly an important way to obtain information on the behavior of the various biomaterials, e.g., their different resorption patterns, bone formation with the use of particles or blocks, tissue response to the possible long-term persistence of some biomaterials. Besides light microscopy, other techniques can be used to evaluate histological slides containing biomaterials, i.e., Scanning Electron Microscopy, Transmission Electron Microscopy, Atomic Force Microscopy, Confocal Laser Scanning Microscopy, and Synchrotron Micro-CT [7,8,9,10,11,12]. These studies have helped in the evolution of bone substitute biomaterials, allowing reduction of morbidity due to the use of autogenous bone grafts, producing biomaterials with properties and physicochemical compositions similar to the host bone tissue. The present retrospective review aimed to evaluate the histological and biological results using different bone substitute biomaterials, in a time period of over three decades.

## 2. Materials and Methods

A retrospective evaluation of the scientific production of the Implant Retrieval Center Laboratory of University “G. D’Annunzio” of Chieti-Pescara in the last three decades was performed with databases PubMed, Scopus, and EMBASE in order to consider only the indexed scientific production of the Laboratory. The papers list has been obtained through the indexed papers lab archive.The articles screened were limited to papers dealing with bone substitute biomaterials for jawbone regeneration. The selected papers underwent a qualitative evaluation, analyzing the different biomaterials used, the study models, sample size, test and control group features, the study timepoints and the experimental findings.

### 2.1. Inclusion Criteria

Articles published up to January 2021 were included without language restriction. The articles screened were limited only to papers dealing with bone substitutes and scaffolds in the form of granules and block grafts for bone regeneration. The scientific articles included were verified for the qualitative analysis. According to the search criteria, human studies, in vitro studies, and animal model studies were evaluated. Articles that did not conform to the inclusion criteria and literature reviews were excluded from the review. The papers included were also categorized into block scaffolds, particulate graft and advanced experimental biomaterials.

### 2.2. Selection of the Studies

**Figure 1:**
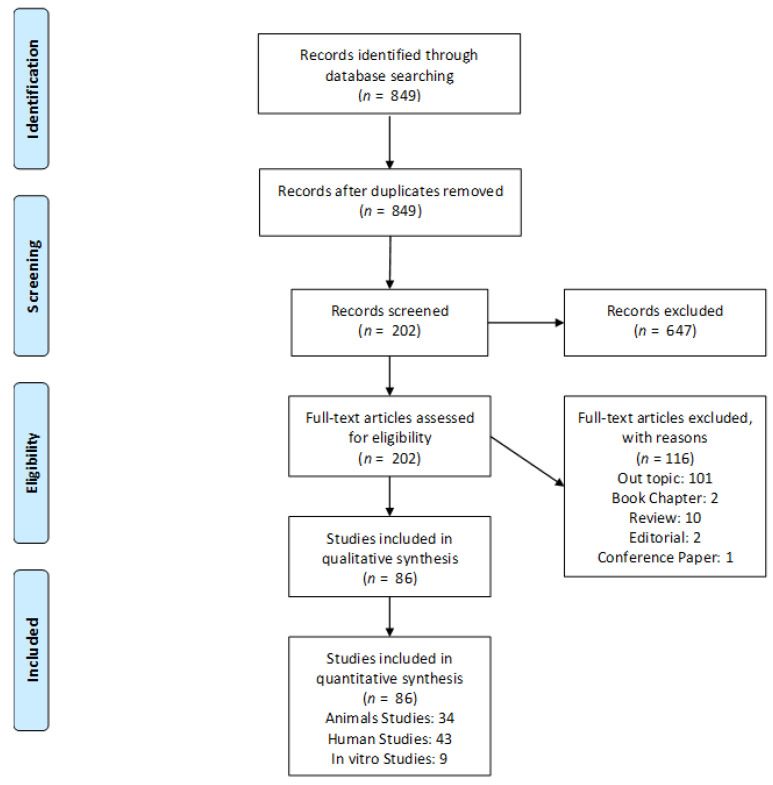
PRISMA flowchart of the included studies.

The experimental data and article selection were conducted independently by two expert reviewers (M.T. and A.P.). They used a particular designed data form by Excel software package (Office Microsoft, Redmond, WA, USA). Therefore, when the abstract was not available, the paper’s full text was obtained and checked. Literature reviews, case reports, and book chapters were excluded from the qualitative analysis. For excluded articles, a description was performed of the reasons for exclusion (Figure 1).

## 3. Results

A total of 86 papers were found and evaluated. Most of the available biomaterials in the past three decades in the market have been studied and were reported, i.e., anorganic bovine bone, equine bone, porcine bone, biphasic calcium-phosphate ceramics, phycogene hydroxyapatite, bio-glass, calcium carbonate, autologous bone, polylactide-polyglycolide, porous hydroxyapatite, beta-tricalcium-phosphate.

### 3.1. Anorganic Bovine Bone (ABB)

In most of the samples, the biomaterial grafted particles were surrounded by newly-formed bone. This newly-formed bone was in close and tight contact with the biomaterial particles’ external surface, and no gaps, no fibrous, connective tissue, or foreign body reaction cells were found at the bone-biomaterial interface. In a few microscopic fields, osteoblasts were observed depositing osteoid matrix directly on the biomaterial surface, and, in other areas, a few osteoclasts could be observed at the interface with the grafted particles (Table 1) [13]. Slow resorption of the particles of ABB has been reported [13,14,15]. A study [16] found that it was possible to generate osteoclasts, starting from the monocytes of peripheral blood, on the surface of slices of ABB, and that these osteoclasts were able to resorb the xenograft. ABB was a highly biocompatible and osteoconductive biomaterial with no foreign body reaction cells, no connective tissue, and no chronic inflammatory processes [14]. Some of the specimens containing ABB were retrieved, due to different causes, after many years [13,15,17,18,19,20]. In all of these cases of long-term persistence of ABB in the tissues, lamellar, mature, compact bone was found at the bone-biomaterial interface, always in close contact with the particles, and, in some specimens under scanning electron microscopy, several projections of newly-formed bone were seen penetrating the ABB particles [17]. Moreover, relatively high concentrations of calcium and phosphorus found in the biomaterial particles decreased gradually toward the interface within the bone [17]. The residual grafted particles had not interfered with the formation of new bone in the site and had not produced any untoward or adverse effects. With the use of several biomaterials in sinus augmentation procedures, histology showed that in human biopsies retrieved after 6 months during implant insertion, the regenerated bone showed, in all cases, a similarity to D3 bone type, and only in a more extended period sample of ABB was the bone tissue comparable to D2 bone type, showing that, with the use of some biomaterials, an increase of bone density over time could occur [21]. Angiogenesis plays a relevant, pivotal role in osteogenesis, and a close temporal and spatial relationship between them has been reported [13,15,17,18,19,20]. Angiogenesis can be evaluated by counting the number of newly-formed small blood vessels (micro-vessel density–MVD) and using immunohistochemistry, e.g., for Vascular Endothelial Growth Factor (VEGF). ABB seemed to be able to induce an increase in MVD that reached a higher value after 6 months (Table 1) [16]. A higher percentage of vessels and cells positive for VEGF were found in areas where there was newly-formed bone [21]. In a human study comparing autologous bone (AB) and ABB in sinus augmentation procedures, it was found that the difference in MVD and VEGF expression between sinuses augmented with AB and ABB was statistically significant, with higher values in AB specimens [19]. Similar results were found in another paper [16], with the highest values of MVD and VEGF expression in sites grafted with AB. In another human study on maxillary ridge defects, both sides augmented with AB and ABB presented a higher and statistically significant quantity of MVD compared to control, non-augmented sites [3]. Molecular studies found that ABB did not enhance the production of proinflammatory cytokines [21] and that the up-and down-regulation of several different genes could explain the reported bio-affinity of ABB for host tissues, its biological affinity to osteogenic cells, and its capability to stimulate osteogenic differentiation (Table 1) [21].

### 3.2. Porcine Bone (PB)

Dual-phase porcine xenografts have different properties according to their composition and processing. Two different categories can be defined based on the varieties of bone present within the graft:

1. collagenated cortico-cancellous porcine bone
2. collagenated cortical porcine bone

Both families undergo a manufacturing process which preserves the main organic phase, represented by Collagen I protein, and prevents the ceramicization of the biomaterial which would limit the biological properties of the graft (Table 2). Most studies performed on collagenated cortico-cancellous porcine bone found that grafted particles were surrounded by newly-formed bone starting as early as 3 months of healing [1,3,8,23,24]. Morphometric data, as extracted by histology and microCT analysis, conducted on post-extraction sockets, treated with collagenated cortico-cancellous heterologous pre-hydrated bone mix revealed a greater number of trabeculae filling the defect, compared to the spontaneously healed bone control samples, suggesting an improved strength of the socket, with histology showing the amount of biomaterial decreasing over time and replaced with newly formed bone. In contrast, less dense bone with wide marrow spaces was found in control samples. All data converge to confirm the good performance of collagenated cortico-cancellous porcine bone as substitute for the preservation of human maxillary (Table 2) [8]. Clinical and histological outcomes indicated that collagenated cortico-cancellous porcine bone graft was found to be a highly biocompatible and osteo-conductive biomaterial that, thanks to its elevated interconnecting micro-porosity, could be used with success, alone or in association with autologous bone, in sinus augmentation procedures (Table 2) [23] A synchrotron study supports and validates the collagenated Cortico-Cancellous Porcine Bone graft capability of osteo-conduction, offering adequate support for tissue reconstruction, due to its biological characteristics and ability to support cell growth and differentiation [24]. In addition, the microCT analysis revealed a gradual decrease of the porcine graft biomaterial starting from the first week of culture, with the residual grafted particles not interfering with the formation of new bone in the site and without producing any untoward or adverse effects (Table 2) [24].

An experimental study found that collagenated cortico-cancellous porcine bone granules embedded with growth factors (bFGF, VEGF etc.), derived from mesenchymal stem cells (MSCs) could promote an increase in new bone formation, in close and tight contact with the biomaterial particles’ external surface, and stimulate vascularization in a rat calvarial defect model, without any inflammatory cell infiltration at the bone-biomaterial interface [25]. Collagenated cortico-cancellous porcine bone graft therefore can be considered a good reservoir for growth factor in a bioactive form allowing a good natural delivery system for bone healing. Finally, it was also found through an in vivo experiment that collagenated cortico-cancellous porcine bone mix and pre-hydrated CCCPB mix presented higher biocompatibility and were capable of inducing faster and greater bone formation compared to cancellous block of xenogenic bone [1]. On the other hand, collagenated cortical porcine bone showed no evidence of graft resorption after 4 months healing. The percentage of the residual graft material was the same after 4 and 6 months with no interference with bone regeneration processes and implant osseointegration. A slight increase in newly formed bone was found in the 6-month specimens (31%) as compared to the 4-month (28%) specimens [1]. Mature bone with many osteocytes was observed near the particles, and under Transmission Electron Microscopy all phases of bone formation (osteoid matrix, woven bone, and lamellar bone) were observed. All together these results suggest that collagenated cortical porcine bone substitutes, through their osteo-conductive potential, allow predictable placement of dental implants in the regenerated maxillary premolar and molar areas (Table 2) [25].

### 3.3. Equine Bone (EQ)

Equine bone appeared to be a biocompatible biomaterial associated with new vessel ingrowth (Table 3). These small, newly-formed vessels are always found near and in close association with the advancing front of the new bone formation [26]. Higher intensity of VEGF expression was observed in newly-formed bone, whereas a low VEGF intensity was found in mature, compact, lamellar bone (Table 3) [26]. With the use of equine collagenated blocks, it was found that newly-formed bone was in close contact with the biomaterial [21,28,29,30,31]. An in vitro study, with the use of equine spongy bone slices, reported that osteoclasts could be produced from cells of the peripheral blood and that these cells were able to resorb the biomaterial (Table 3) [26].

### 3.4. Biphasic Calcium Phosphate (BCP)

Biphasic calcium phosphate (BCP) is an alloplastic biomaterial available in different microstructures, micro- and macro-porosities. The BCP particles showed a successful integration with the newly formed bone in mandibular sites [32] and in maxillary sinus augmentation procedures (Table 4) [3]. BCP could be adapted to large jaw defects through the CAD/CAM technique, and this biomaterial has shown a very good bone biocompatibility and osteo-conductivity [24,33]. In a study published many years ago, using a BCP composed of 50% hydroxyapatite and 50% beta-tricalcium-phosphate, it was found that many particles were surrounded by newly-formed bone and that some particles were undergoing resorption processes and were being gradually substituted by newly-formed bone [3]. With the use of BCPs with different percentages of the two constituents (Table 4) (HA and B-TCP), it was found that the particles were always surrounded by newly-formed bone (Table 4).

### 3.5. Calcium Carbonate

The particles were almost always surrounded by mature bone [35,36]. This biomaterial was clinically suitable for sinus augmentation procedures according to a successful new bone formation and graft integration (Table 5) [3,29,35].

The calcium carbonate-derived scaffold and graft could be obtained by coral aragonite or artificially sintered-procedure (Table 5) [3,35,36]. This biomaterial could be subjected to resorption with an higher efficacy then calcium-derived materials [3,35,36]. The graft porosity is able to promote the new bone formation in-growth and remodeling (Table 5) [3,35,36].

### 3.6. Bioglass

Bio-glass was a highly osteoconductive material with the newly-formed bone around all particles, even those located in the central portion of the defects (Table 6) [2,38]. This biomaterial has resulted in being biocompatible and improved new bone formation in maxillary sinus lift [2]. The bio-glass bone substitutes are composed of minerals that are commonly present in the body, with calcium and phosphorous oxides proportions similar to the human bone percentage (Table 6) [39,40]. In literature, the bioglasses demonstrated an increase collagen depositions when in contact with the connective tissues [39]. Moreover, its porosity is able to increase the scaffold properties and the new bone formations when used to fill bone defects producing an in-growth of the osteoid matrix and the newly formed bone [41,42]. On the contrary, this biomaterial could be associated with a low fracture resistance and should be used in regions with no passive loading forces [41]. Different authors reported the antibacterial bio-glass’s property when used for bone regeneration procedures (Table 6) [41].

### 3.7. Porous Hydroxyapatite (Porous HA)

Porous HA can be a suitable synthetic material for sinus augmentation procedures [43]. Biomaterial particles were observed in close and tight contact with mature, compact, and lamellar bone (Table 7) [21,34,43,44,45,46]. A high quantity of newly-formed bone was found [43,47]. A large portion of the biomaterial particles was surrounded by bone [16,19,36,48,49]. Porous HA was reported to be of use also as joint prostheses [15,22,43,50,51]. The use of custom-made scaffolds made of porous HA Blocks has been reported that produced a vertical bone gain of 6.93 ± 0.23 mm after 6 months of healing (Table 8) [43,47].

## 4. Discussion

During all these years of research, different study models were used in our center. The evolution of the evaluation methods followed the progress of the techniques applied to determine the tested materials’ biological quality. However, the methods most used in in vivo and clinical studies were histological and histomorphometric assessments of newly formed bone tissue. Large parts of these tested biomaterials have helped their implantation in the market or have evaluated those already available [3,23,62,63]. An important aspect is determined by the different origin of the xenogenic bone graft when used in bone regeneration procedure. Scarano et al. reported no significant differences between equine and porcine cortico/cancellous graft when used on standardized iliac defect [1]. Moreover, the authors reported a more highly significant new bone formation in grafted sites compared to the control empty bone defect [1]. The main characteristics observed, mainly in experimental studies, were not only the formation of bone tissue or the contact of the new tissue with the bone substitute but also the reabsorption of the material implanted in the cells present around the biomaterial (e.g., macrophages, giant cells multinucleate, osteoblasts, osteoclasts, and osteocytes). The impact produced by the material on the implanted tissue could identify the necessity for structural modifications (i.e., composition, granulation, and sintering) [43,64]. Thus, it is possible to improve the bone substitute for subsequent application in humans. In this long period, studies were made in granules and block formats, materials of different structures, but both of great clinical importance, mainly acting as a scaffold, the materials having osteo-conductivity as their main characteristic. Clinically, granules are most often used for small bone defects (e.g., dental socket), while blocks are reserved for larger areas (e.g., horizontal augmentation). The surgeon needs to take into account the structural and physicochemical characteristics of biomaterials. On the contrary, the majority of the evaluated graft biomaterials have shown a slow resorption, and the presence of residual grafted particles were found many years after the grafting procedure. [13,14,15,65,66]. This fact could be advantageous when the stability of the bone graft could be essential for the success of the regeneration, such as in sinus augmentation procedures (for helping in the contrast with repneumatization of the maxillary sinuses), in alveolar socket preservation techniques, and in severe mandibular atrophies [62,67,68]. Another advantageous effects is determined by the antibacterial role of some biomaterials and bioglasses, that could represent a useful strategy also for infected sites grafting in order to protect the healing phases of the bioscaffold osseointegration [69]. An in vitro study [70] found that it was possible to generate osteoclasts, starting from the monocytes of peripheral blood, on the surface of slices of ABB, and that these osteoclasts were able to resorb the xenograft. Many different advanced bioscaffold constructs have been studied such as graphene oxide-biomaterials, platelet derived growth factors/β-TCP constructs, interconnected porous hydroxy-apatite complex, rhBMP-7/deproteinised bone substitute, and autologous osteoblasts/polymeric scaffolds [20,28,45,54,55,56,57,58,59,60,71]. Innovations correlated with new bone substitutes, such as rh-BMP and/or the incorporation of materials such as collagen, seeking improvement by bringing the ability of osteo-induction to improve the quality of the material presented, wer3 also studied, showing promising results, mainly the incorporation of collagen, which helps in the formation of the initial bone matrix and helps the arrival and adhesion of osteoprogenitor cells [24]. Concerning BMPs and mesenchymal cells, both are currently used in some countries in clinical procedures. However, it is possible to observe some studies that show limitations of these materials, either due to the exacerbation of bone tissue newly formed by BMPs or the formation of teratomas/hamartomas by mesenchymal cells in the region where these materials are implanted. More studies related to these materials are needed [24]. Among the studied materials, histological responses presented by the presented materials, mainly xenogenous and alloplastic, were excellent, considered safe materials, and capable of acting properly to reconstruct the new bone tissue [24]. However, they are matrices that will only assist in bone conduction. It is interesting to incorporate other components in these biomaterials, which may benefit the bone tissue into which they are implanted.

## 5. Conclusions

Currently, the search for biomaterials that will present properties similar to autogenous grafts is constant. The slow resorption rate of xeno-genic biomaterials could be useful when a higher bone graft stability is clinically advantageous for a successful dental implant positioning. After thirty years of research with bone substitutes, their safety and long-term effectiveness have been demonstrated. However, no biomaterial evaluated presented the same characteristics of the autologous bone. On the other hand, the use of xeno-genous or alloplastic grafts has been shown to be an excellent and safe option.

## Figures and Tables

**Table 1:** Summary table of the anorganic bovine bone (ABB) findings of the papers included.

Authors	Study Findings	Results	Biomaterials and Methodologies Characteristics	Study Model Model	Sample Size	Defect	Test Group	Control Group	Timepoints
Traini et al., Clin Implant Dent Relat Res. 2015 [21]	in the same experimental time, equine group specimens showed evident resorption phenomena,	no or little signs of resorption were evident in the porcine group specimens.	sinus augmentation	Human	295 patients	Maxillary sinus	Anorganic bovine bone (ABB) Dense hydroxyapatite (dHA) DAC Porous hydroxyapatite (porHA) Cortical/cancellous porcine bone (cortPB) Macroporous biphasic calcium phosphate (Ca_2_PO_4_); Demineralized freeze-dried bone allograft (DFDBA) Calcium carbonate (CaCO_3_); Polymer of polylactic and polyglycolide acids (PLL/PLG) Anorganic bovine bone with synthetic peptide P-15 (P-15) PepGen P-15™; sulphate (CaSO_4_) Surgiplaster sinus;	-	6 months
Testori et al., Int J Periodontics Restorative Dent. 2012 [16]	excellent properties of particular hydroxyapatite porous microstructure with a high percentage of interconnected micropores that promote the ingrowth of osteogenic cells and vessels, making graft integration easier and faster.	Histomorphometry showed that the percentages of newly formed bone, residual grafted particles, and marrow spaces were 25.1 ± 2.3%, 37.3 ± 1.1%, and 38.5 ± 3.1%, respectively.	Histological and histo—morpho--metrical analysis	human	1 case bilaterally	split case	High temperature-treated bovine porous hydroxyapatite	-	9 months
Degidi et al., J Oral Implantol. 2013 [13]	Implant placement into extraction sockets can result in favorable radiological results even in the presence of evident alterations of the buccal bone wall.	The higher and lower intensities of vascular endothelial growth factor and NOS3 expression were prevalent in the sites grafted with autologous bone with significant differences with the controls (*p* < 0.05).	Histological and histo—morpho--metrical analysis	human	1 patients, 2 sites	split case	Anorganic bovine bone	anorganic bovine matrix added to a cell-binding peptide (PepGen P-15)	8 years
Iezzi et al., Clin Oral Implants Res. 2012 [3]	within the limitations of the present study, the data provided support the fact that all these biomaterials can be used, successfully, in sinus augmentation procedures.	Histomorphometry showed that, in all biomaterials, newly formed bone and residual grafted material particles represented about 30%.	Histological and histo—morpho--metrical analysis	human	15 patients 30 sinuses, 82 implants	split cases	phycogene hydroxyapatite, biphasic calcium phosphate ceramics, calcium carbonate, porcine bone and anorganic bovine bone	-	6 months
Chackartchi Clin Oral Implants Res. 2011 [19]	Both sizes of BBM granules preformed equally and achieved the aim of the sinus floor augmentation procedure clinically and histologically.	Histo-morpho-metric analysis revealed that both granule sizes produced the same pattern of bone formation,	Histological and histo-morpho-metrical analysis	human	10 patients/20 sinuses	split cases	two different particle sizes of bovine bone mineral (BBM)	-	6 months
Traini et al., J Periodontol 2007 [22]	The tissue pattern appeared composed by residual ABB particles in close contact to the newly formed bone. The bone mineralized matrix around the ABB had collagen fibers randomly oriented and more osteocytes embedded. The results demonstrate both a high level of osteo-conductivity and a “biomimetic” behavior over the long term.	We observed a mean amount of newly formed bone of 46.0 ± 4.67%, ABB remnants of 16.0 ± 5.89%, and marrow spaces of 38.0 ± 8.93%. The osteocyte index was 4.43 for bone around ABB and 3.27 in the trabecular bone at a distance from the particles.	Histological and histo-morpho-metrical analysis	human	Case Report	Sinus Augmentation	anorganic bovine bone	-	6 months
Orsini et al., Oral Dis 2007 [15]	Bio-Oss particles did not interfere with bone-healing processes after sinus augmentation procedures and promoted new bone formation. This study can help clinicians to understand better the morphological characteristics of bone regeneration processes using Bio-Oss after 20 months and, most importantly, after a longer	Under transmission electron microscopy, it was possible to characterize the bone-biomaterial interface; in the 20-month specimen an electron-dense layer was seen, whereas, almost no electron-dense lines were seen at the interface in the 7-year specimen.	Histological and histo-morpho-metrical analysis, TEM	human	Case Report	Sinus Augmentation	anorganic bovine bone + collagen membrane	-	6 months
Carinci et al., Arch Oral Biol. 2006 [18]	he data reported are, to our knowledge, the first genetic portrait of Bio-Oss effects. They can be relevant to our improved understanding of the molecular mechanism underlying bone regenerative procedures and as a model for comparing other materials with similar clinical effects.	The log2 ratios for all the targets on the array were then calibrated using the normalization factor, and log2 ratios outside the 99.7% confidence interval (the median 3 times S.D. = 0.52) were determined as significantly changed in the treated cells.	Gene expression Microarray	osteoblast-like MG63 cells	In vitro study	Cells culture	anorganic bovine bone	Not treated cells	-
Orsini et al., J Biomed Mater Res B Appl Biomater. 2005 [14]	The analyses showed that Bio-Oss particles do not interfere with the normal osseous healing process after sinus lift procedures and promote new bone formation.	newly formed compact bone was present. In the first bone lamella collagen fibers contacting the Bio-Oss surface were oriented at 243.73 ± 7.12 degrees (mean ± SD), while in the rest of the lamella they were oriented at 288.05 ± 4.86 degrees (mean ± SD) with a statistically significant difference of 44.32 degrees (*p* < 0.001).	Histological and histo-morpho-metrical analysis, TEM, SEM	12 patients	Human	Sinus Augmentation	anorganic bovine bone	-	6 months
Corinaldesi et al., Br J Oral Maxillofac Surg. 2013 [20]	In this pilot controlled trial of the use of rhBMP-7, histological analyses showed that it resulted in the formation of less bone than treatment with inorganic bovine hydroxyapatite.	Histological and histo-morpho-metric analyses of biopsy specimens showed that there was significantly more new bone on the control side (19.9 (6.8)%) than on the test side (6.6 (4.8)%).	Histological histo-morphometry	Human	9 patients/18 sinuses	Maxillary sinus	rhBMP-7 (Osigraft) with deproteinized bone substitute (0.5 g on the test side)	deproteinized bone alone (2.0 g on the control side)	6 months

**Table 2:** Summary table of the porcine bone (PB) findings of the papers included.

Authors	Study Findings	Results	Biomaterials and Methodologies Characteristics	Study Model Model	Sample Size	Defect	Test Group	Control Group	Timepoints
Mijiritsky et al., Material 2017 [25]	The controlled release of active growth factors from porcine bone granules can enhance and promote bone regeneration.	The higher and lower intensities of vascular endothelial growth factor and NOS3 expression were prevalent in the sites grafted with autologous bone with significant differences with the controls (*p* < 0.05).	In vitro MCS Stem cells + Bone porcine granules activity	Rat	12	Calvarial defects	MCS Stem cells + Bone porcine granules	Native bone granules	1 h, 6, 12, 24 h, 3 and 7 days. (in vitro)
Giuliani et al., Clin Oral Investig. 2018 [8]	MicroCT revealed that in the grafted sites there were a greater number of trabeculae,	Increase of the SV/TV and of the SNr, with a significant growth from 3 to 6 months from grafting (SV/TV: *p* = 0.003; SNr: *p* < 0.001) could be observed.	Porcine Bone MP3 in extraction sockets	Human	28	Porcine Bone MP3 in extraction sockets	Porcine Bone MP3 in extraction sockets	Unfilled	12 months
Scarano et al., Biomed res. 2016 [1]	these data suggest that these biomaterials have higher biocompatibility and are capable of inducing faster and greater bone formation.	SEM-EDS analysis showed a Ca/P ratio of 1.8 for BO, 2.2 for EP, and 1.5 for P-15. Under CPLM, BO showed no significant difference for transverse (18.4 ± 2.7%) and longitudinal (16.3 ± 1.8%) bone collagen fibers (*p* = 0.195);	GBR in iliac sheep crest	sheep	4 animals	peri implant defects	Porcine cortico-cancellous mix: Equine blocks: Porcine collagenated.	-	4 months
Cassetta et al., Clin Oral Implants Res. 2015 [23]	The clinical and histological results of this study indicated that porcine bone alone or in combination with autologous bone are biocompatible and osteoconductive materials and can be successfully used in sinus augmentation procedures.	Histomorphometry showed that the percentage of newly formed bone was 35.2 ± 3.6%, marrow spaces 35.6 ± 2.3%, and residual grafted material 37.1 ± 3.8%.	Human	Human	10 patients	Maxillary sinus	100% autologous bone (Group A), 100% porcine bone (Group B), and a 50:50 mixture of autologous and porcine bone (Group C)	-	2 months
Tetè J Craniofac Surg. 2014 [26]	a more rapid and intense vascularization was achieved in equine bone substitute group, as demonstrated by immunohistochemical analysis for VEGF expression.	The higher and lower intensities of vascular endothelial growth factor and NOS3 expression were prevalent in the sites grafted with autologous bone	sinus augmentation	Human	10 patients	Maxillary sinus	equine bone, porcine bone	-	6 months
Barone et al., J Periodontol. 2014 [27]	Porcine bone alone or in combination with autologous bone are biocompatible and osteoconductive materials and can be successfully used in sinus augmentation procedures.	Osteoblast grown on Bio-Oss showed a normal RNA expression of osteo--nectin, integrin beta1 and PDGF.	Socket Preservation	Human	64 patients	Post extractive socket	Flapless	full-thickness mucoperiosteal flap	2 weeks
Traini et al., Clin Implant Dent Relat Res. 2015 [21]	in the same experimental time, equine group specimens showed evident resorption phenomena,	no or little signs of resorption were evident in the porcine group specimens.	sinus augmentation	Human	295 patients	Maxillary sinus	Anorganic bovine bone (ABB) Dense hydroxyapatite (dHA) DAC Porous hydroxyapatite (porHA) Cortical/cancellous porcine bone (cortPB) Macroporous biphasic calcium phosphate (Ca_2_PO_4_); Demineralized freeze-dried bone allograft (DFDBA) Calcium carbonate (CaCO_3_); Polymer of polylactic and polyglycolide acids (PLL/PLG) Anorganic bovine bone with synthetic peptide P-15 (P-15) PepGen P-15™; sulphate (CaSO_4_) Surgiplaster sinus;	-	6 months
Iezzi et al., Clin Oral Implants Res. 2012 [3]	within the limitations of the present study, the data provided support the fact that all these biomaterials can be used, successfully, in sinus augmentation procedures.	Histomorphometry showed that, in all biomaterials, newly formed bone and residual grafted material particles represented about 30%.	Histological and histo-morpho-metrical analysis	human	15 patients 30 sinuses, 82 implants	split cases	phycogene hydroxyapatite, biphasic calcium phosphate ceramics, calcium carbonate, porcine bone and anorganic bovine bone	-	6 months

**Table 3:** Summary table of the equine bone (EQ) findings of the papers included.

Authors	Study Findings	Results	Biomaterials and Methodologies Characteristics	Study Model Model	Sample Size	Defect	Test Group	Control Group	Timepoints
Scarano et al., Biomed res. 2016 [1]	these data suggest that these biomaterials have higher biocompatibility and are capable of inducing faster and greater bone formation.	SEM-EDS analysis showed a Ca/P ratio of 1.8 for BO, 2.2 for EP, and 1.5 for P-15. Under CPLM, BO showed no significant difference for transverse (18.4 ± 2.7%) and longitudinal (16.3 ± 1.8%) bone collagen fibers (*p* = 0.195);	GBR in iliac sheep crest	sheep	4 animals	peri implant defects	Porcine cortico-cancellous mix: Equine blocks: Porcine collagenated.	-	4 months
Tetè J Craniofac Surg. 2014 [26]	a more rapid and intense vascularization was achieved in equine bone substitute group, as demonstrated by immunohistochemical analysis for VEGF expression.	The higher and lower intensities of vascular endothelial growth factor and NOS3 expression were prevalent in the sites grafted with autologous bone	sinus augmentation	Human	10 patients	Maxillary sinus	equine bone, porcine bone	-	6 months
Traini et al., Clin Implant Dent Relat Res. 2015 [21]	in the same experimental time, equine group specimens showed evident resorption phenomena,	no or little signs of resorption were evident in the porcine group specimens.	sinus augmentation	Human	295 patients	Maxillary sinus	Anorganic bovine bone (ABB) Dense hydroxyapatite (dHA) DAC Porous hydroxyapatite (porHA) Cortical/cancellous porcine bone (cortPB) Macroporous biphasic calcium phosphate (Ca_2_PO_4_); Demineralized freeze-dried bone allograft (DFDBA) Calcium carbonate (CaCO_3_); Polymer of polylactic and polyglycolide acids (PLL/PLG) Anorganic bovine bone with synthetic peptide P-15 (P-15) PepGen P-15™; sulphate (CaSO_4_) Surgiplaster sinus;	-	6 months
Tete et al., Eur J Histochem. 2013 [29]	It can be concluded that calcium carbonate was shown to be clinically suitable for sinus elevation procedures after 1 to 5 years of follow-up and histologically biocompatible and osteoconductive.	Histomorphometry showed that the percentage of newly formed bone was 35.2 ± 3.6%, marrow spaces 35.6 ± 2.3%, and residual grafted material 37.1 ± 3.8%.	sinus augmentation	Human	20 patients	Maxillary sinus	equine bone,	autologous	6 months
Artese et al., Implant Dent. 2011 [30]	The results obtained showed that the mixture of autologous and equine bone was biocompatible	The higher and lower intensities of vascular endothelial growth factor and NOS3 expression were prevalent in the sites grafted with autologous bone with significant differences with the controls (*p* < 0.05).	Histological and histo-morpho-metrical analysis	human	16 patients	split cases	autologous and equine bone	-	6 months
Perrotti et al., Clin Oral Implants Res. 2009 [31]	This study enables clinicians to tailor the usage of equine spongy bone and presents a model, which can be applied to the preclinical assessment of bone substitute material’s resorbability and resorption rates.	cells were functionally active on equine spongy bone with statistically significant differences compared with the control in the release of tartrate-resistant acid phosphatase (TRAcP5b) at days 14 and 21 of culture.	RT PCR	In vitro culture	Peripheral blood mononuclear cells	Human osteoclasts (OCLs)	equine spongy bone	-	21 days

**Table 4:** Summary table of the Biphasic calcium phosphate (BCP) and Beta Tri-calcic Phosphate (Beta-TCP) findings of the papers included.

Authors	Study Findings	Results	Biomaterials and Methodologies Characteristics	Study Model Model	Sample Size	Defect	Test Group	Control Group	Timepoints
Mangano Int J Oral Maxillofac Implants. 2013 [32]	the mixture of HA and autogenous bone graft showed lower degree of resorption and higher dimensional stability when compared with autogenous bone graft alone, at least at 180 days of healing.	The higher and lower intensities of vascular endothelial growth factor and NOS3 expression were prevalent in the sites grafted with autologous bone with significant differences with the controls (*p* < 0.05).	sinus augmentation	Human	12 sites	Maxillary sinus	Macro-porous biphasic-calcium phosphate (MBCP) comprising hydroxyapatite/tricalcium phosphate (HA/TCP) 60/40	-	6 months
Scarano et al., Int J Oral Maxillofac Implants. 2012 [34]	Data from the preliminary results demonstrated that MBCP is a biocompatible and osteoconductive material that can be successfully used as a grafting material for sinus floor augmentation.	Histologic investigation showed that the macro-porous biphasic calcium phosphate grafted particles were embedded and integrated in the newly formed bone; this bone was in close and tight contact with the biomaterial particles.	Histological and histo-morpho-metrical analysis	rabbit	6 animals, 24 specimens	rabbit tibiae	algae-derived hydroxyapatite	-	4 weeks
Iezzi et al., Clin Oral Implants Res. 2012 [3]	within the limitations of the present study, the data provided support the fact that all these biomaterials can be used, successfully, in sinus augmentation procedures.	Histomorphometry showed that, in all biomaterials, newly formed bone and residual grafted material particles represented about 30%.	Histological and histo-morpho-metrical analysis	human	15 patients 30 sinuses, 82 implants	split cases	phycogene hydroxyapatite, biphasic calcium phosphate ceramics, calcium carbonate, porcine bone and anorganic bovine bone	-	6 months
Giuliani et al., Implant Dent. 2016 [24]	The scaffold morphology was confirmed to influence the long-term kinetics of bone regeneration. Considering the whole mineralized	Large amount of newly formed bone was detected in the retrieved specimens, together with a good rate of biomaterial resorption and the formation of a homogeneous and rich net of new vessels.	Synchrotron Radiation X-ray Microtomography	Maxillary sinus	14 subjects	Block vs particles Tri-calcic Phosphate Beta	-	8 months	9 months
Mangano et al., Clin Oral Implants Res 2015 [33]	The findings indicated a high biocompatibility and osteo-conductivity of HA-beta-TCP 30/70, for sinus augmentation procedures	The histomorphometric analysis revealed 26 ± 2% of residual grafted biomaterial, 29 ± 3% of newly formed bone, and 45 ± 2% of marrow spaces.	Histological and histo-morpho-metrical analysis	human	12 patients	Sinus Augmentation	beta-TCP 30/70	-	6 months

**Table 5:** Summary table of the Calcium carbonate findings of the papers included.

Authors	Study Findings	Results	Biomaterials and Methodologies Characteristics	Study Model Model	Sample Size	Defect	Test Group	Control Group	Timepoints
Mangano et al., Int J Periodontics Restorative Dent. 2014 [35]	calcium carbonate was shown to be clinically suitable for sinus elevation procedures after 1 to 5 years of follow-up and histologically biocompatible and osteoconductive.	The osteoclast-like cells preferred the small-size BBM particles and not the large particles both in the small-size and the large-size granules group.	sinus augmentation	Human	24 patients, 68 implants	Maxillary sinus	calcium carbonate	-	1–5 years
Tete et al., Eur J Histochem. 2013 [29]	It can be concluded that calcium carbonate was shown to be clinically suitable for sinus elevation procedures after 1 to 5 years of follow-up and histologically biocompatible and osteoconductive.	Histomorphometry showed that the percentage of newly formed bone was 35.2 ± 3.6%, marrow spaces 35.6 ± 2.3%, and residual grafted material 37.1 ± 3.8%.	sinus augmentation	Human	20 patients	Maxillary sinus	equine bone,	autologous	6 months
Iezzi et al., Clin Oral Implants Res. 2012 [3]	within the limitations of the present study, the data provided support the fact that all these biomaterials can be used, successfully, in sinus augmentation procedures.	Histomorphometry showed that, in all biomaterials, newly formed bone and residual grafted material particles represented about 30%.	Histological and histo-morpho-metrical analysis	human	15 patients 30 sinuses, 82 implants	split cases	Phyco-gene hydroxyapatite, biphasic calcium phosphate ceramics, calcium carbonate, porcine bone and anorganic bovine bone	-	6 months
Pettinicchio et al., Aust Dent J. 2012 [37]	the clinical use of heterologous particulate equine-derived biomaterial may ensure long-term predictability of implant-prosthetic rehabilitation	Osteoblast grown on Bio-Oss showed a normal RNA expression of osteo-nectin, integrin beta1 and PDGF.	Scanning electron microscopy (SEM) and energy dispersive X-ray spectroscopy (EDS)	human	6 specimens	Maxillary sinus	calcium sulphate	-	6 months

**Table 6:** Summary table of the Bio-glass findings of the papers included.

Authors	Study Findings	Results	Biomaterials and Methodologies Characteristics	Study Model Model	Sample Size	Defect	Test Group	Control Group	Timepoints
Scarano et al., Implant Dent. 2006 [2]	All biomaterials examined resulted in being biocompatible and seemed to improve new bone formation in maxillary sinus lift. No signs of inflammation were present. The data are very encouraging because of the high number of successfully treated patients and the good quality of bone found in the retrieved specimens.	Some biomaterials were more resorbable than others. Included are the histomorphometry clarified features of the newly formed bone around the different grafted particles.	Histological and histo-morpho-metrical analysis	human	94 patients	Sinus Augmentation	demineralized freeze-dried bone allograft Biocoral [Inoteb, St. Gonnery, France], Bio-glass [US Biomaterials, Alachua, FL], Fisiograft [Ghimas, Bologna, Italy], PepGen P-15 [Dentsply Friadent CeraMed, Lakewood, CO], calcium sulfate, Bio-Oss [Geistlich Pharma AG, Wohlhusen, Switzerland]	autologous bone,	6 months
Giuliani Clin Implant Dent Relat Res. 2014 [38]	A full-thickness mucoperiosteal flap gave significantly more negative results than that of the less-demanding flapless procedure, with an increased width resorption of the post-extraction site.	Histo-morpho-metric analysis revealed that both granule sizes produced the same pattern of bone formation, surrounding the graft granules, and producing a shape of a network, “bridging” between the BBM particles.	Posterior jaws defect	Human	12 patients	Jaws	coralline-derived (bio-coral) scaffold grafts	Beta-tricalcium phosphate and biphasic-calcium phosphate	6 months
Piattelli et al., J Oral Implantol. 2000 [29]	BG seems to be a highly osteoconductive material.	In control sites, bone was observed only in the peripheral areas of the defects, while in test sites, newly formed bone was found around all BG particles, even those located in the central portion of the defect.	Histological histo-morphometry	rabbits	9 animals	tibial metaphysis	Bio-glass (BG)	Empty defects	4 weeks

**Table 7:** Summary table of the Porous hydroxyapatite (Porous HA) findings of the papers included.

Authors	Study Findings	Results	Biomaterials and Methodologies Characteristics	Study Model Model	Sample Size	Defect	Test Group	Control Group	Timepoints
Bechara et al., Ann Anat 2015 [44]	both intra-oral autologous bone and ncHA may be elected as inter-positional grafting materials to vertically augment posterior atrophic mandibles.	Bone density and marrow spaces were similar between groups. Correlations between the ISQ values and the histometric variables were not observed (*p* > 0.05).	Human	Human	12 patients	Posterior mandible	test group that received an inter-positional inlay resorbable non-ceramic hydroxyapatite	Inter-positional inlay autologous bone graft	8 months + impant placement
Traini et al., Clin Implant Dent Relat Res. 2015 [21]	in the same experimental time, equine group specimens showed evident resorption phenomena,	no or little signs of resorption were evident in the porcine group specimens.	sinus augmentation	Human	295 patients	Maxillary sinus	Anorganic bovine bone (ABB) Dense hydroxyapatite (dHA) DAC Porous hydroxyapatite (porHA) Cortical/cancellous porcine bone (cortPB) Macroporous biphasic calcium phosphate (Ca_2_PO_4_); Demineralized freeze-dried bone allograft (DFDBA) Calcium carbonate (CaCO_3_); Polymer of polylactic and polyglycolide acids (PLL/PLG) Anorganic bovine bone with synthetic peptide P-15 (P-15) PepGen P-15™; sulphate (CaSO_4_) Surgiplaster sinus;	-	6 months
Scarano et al., Oral Maxillofac Surg. 2012 [52]	that phycogene hydroxyapatite can be used, successfully, for sinus augmentation procedures.	Histomorphometry showed that the percentage of newly formed bone was 35.2 ± 3.6%, marrow spaces 35.6 ± 2.3%, and residual grafted material 37.1 ± 3.8%.	Histological and histo-morpho-metrical analysis	human	10 patients	split cases	phycogene hydroxyapatite	-	6 months
Mangano et al., J Oral Implantol. 2006 [43]	After a mean 3 years after implantation, all implants are clinically in function and no surgical or prosthetic complications have occurred. Under light microscopy, newly formed bone was 38.5 ± 4.5%, whereas the residual biomaterial represented 12 ± 2.3% and the marrow spaces represented 44.6 ± 4.2%.	Bone was closely apposed to the biomaterials particles as shown in light microscopy and transmission electron microscopy.	Histological and histo-morpho-metrical analysis	human	24 subjects	Sinus Augmentation	Porous hydroxyapatite (HA)	-	6 months
Doi et al., PLoS ONE. 2012 [45]	IPCHA/implant complex might be able to achieve both bone reconstruction and implant stability. implant/interconnected porous hydroxyapatite complex as new concept graft material.	The ISQs of complex groups was 77.8 ± 2.9 in the 6-month, 72.0 ± 5.7 in the 3-month and 47.4 ± 11.0 in the 2-month. The BICs of complex groups was 2.18 ± 3.77 in the 2-month, 44.03 ± 29.58 in the 3-month, and 51.23 ± 8.25 in the 6-month.	ISQ measurement, histology	dog femur	4 animals	jaws defects	implant/interconnected porous hydroxyapatite complex	implants were placed directly into the femur without any bone substrate.	2, 6 months
Scarano et al., Int J Mol Sci. 2018 [46]	composite sticky graft block increased the mechanical properties	Histomorphometry showed that the percentage of newly formed bone was 35.2 ± 3.6%, marrow spaces 35.6 ± 2.3%, and residual grafted material 37.1 ± 3.8%.	Bone Graft Compressive Loading Test	In Vitro	30	-	APL + graft, Blood + Graft, Physiologic Water + Graft	-	-
Cosso et al., Clin Oral Implants Res. 2014 [48]	Bone density and marrow spaces were similar between groups.	EP showed a significant difference between transverse (4 ± 0.7%) and longitudinal (7.6 ± 2.5%) bone collagen fibers (*p* = 0.015);	sinus augmentation	Human	10 patients, 20 sinus augmentation	Maxillary sinus	autogenous bone and the mixture of hydroxyapatite	autogenous bone	15–180 days
Degidi et al., Clin Oral Implants Res. 2013 [53]	None of the evaluated biomaterials seemed to be ideal.	BO showed no significant difference for transverse (18.4 ± 2.7%) and longitudinal (16.3 ± 1.8%) bone collagen fibers (*p* = 0.195);	Cone-Beam Computed Tomography (CBCT) assessment	Human	69 implant	jaws 15/25 site	Bio-Oss(^®^) collagen graft:	-	12 months
Testori et al., Int J Periodontics Restorative Dent. 2012 [16]	Excellent properties of particular hydroxyapatite porous microstructure with a high percentage of interconnected micropores that promote the ingrowth of osteogenic cells and vessels, making graft integration easier and faster.	Histomorphometry showed that the percentages of newly formed bone, residual grafted particles, and marrow spaces were 25.1 ± 2.3%, 37.3 ± 1.1%, and 38.5 ± 3.1%, respectively.	Histological and histo-morpho-metrical analysis	human	1 case bilaterally	human	High temperature-treated bovine porous hydroxyapatite	-	9 months
Degidi et al., J Oral Implantol. 2013 [13]	Implant placement into extraction sockets can result in favorable radiological results even in the presence of evident alterations of the buccal bone wall.	The higher and lower intensities of vascular endothelial growth factor and NOS3 expression were prevalent in the sites grafted with autologous bone with significant differences with the controls (*p* < 0.05).	Histological and histo-morph-ometrical analysis	human	1 patients, 2 sites	split case	Anorganic bovine bone	anorganic bovine matrix added to a cell-binding peptide (PepGen P-15)	8 years
Chackartchi Clin Oral Implants Res. 2011 [19]	Both sizes of BBM granules preformed equally and achieved the aim of the sinus floor augmentation procedure clinically and histologically.	Histo-morpho-metric analysis revealed that both granule sizes produced the same pattern of bone formation,	Histological and histo-morpho-metrical analysis	human	10 patients/20 sinuses	split cases	two different particle sizes of bovine bone mineral (BBM)	-	6 months
Pettinicchio Clin Oral Investig. 2012 [36]	Bio-Oss^®^ (BO), Engipore^®^ (EP), and PepGen P-15^®^ (P-15). BO particles appeared perfectly osseo-integrated in the trabecular bone.	EP showed a significant difference between transverse (4 ± 0.7%) and longitudinal (7.6 ± 2.5%) bone collagen fibers (*p* = 0.015);	Histological and histo-morpho-metrical analysis	human	20 patients	human	Bio-Oss^®^ (BO), Engipore^®^ (EP), and PepGen P-15^®^ (P-15)	-	6 months
Amerio et al., Clin Oral Implants Res. 2010 [49]	Our findings further support the evidence that Bio-Oss is an excellent biomaterial that does not enhance the production of proinflammatory cytokines.	Compared with control osteoblasts it showed a reduced expression of BSP, BMP-2 and BMP-7, IL-6 and TNF-alpha.	RT PCR	In Vitro	Cell cultures	In vitro	Bio-Oss^®^ (BO) + osteoblast	-	7, 14, 21 days
Iezzi et al., J Periodontol 2007 [50]	Vital, mature bone was formed and maintained over a long period with no chronic inflammatory cell infiltrate, foreign body response, or other adverse effects.	Histomorphometry showed that the mean amount of mature, compact bone was 71.0 ± 2.28%, the mean amount of ABM was 22.1 ± 3.18%, and the mean amount of marrow spaces was 11.2 ± 5.42%.	Histological and histo-morpho-metrical analysis	human	Case Report	Sinus Augmentation	Anorganic bone matrix	-	6 months
Traini et al., J Periodontol 2007 [22]	The tissue pattern appeared composed by residual ABB particles in close contact to the newly formed bone. The bone mineralized matrix around the ABB had collagen fibers randomly oriented and more osteocytes embedded. The results demonstrate both a high level of osteo-conductivity and a “biomimetic” behavior over the long term.	We observed a mean amount of newly formed bone of 46.0 ± 4.67%, ABB remnants of 16.0 ± 5.89%, and marrow spaces of 38.0 ± 8.93%. The osteocyte index was 4.43 for bone around ABB and 3.27 in the trabecular bone at a distance from the particles.	Histological and histo-morpho-metrical analysis	human	Case Report	Sinus Augmentation	anorganic bovine bone	-	6 months
Orsini et al., Oral Dis 2007 [15]	Bio-Oss particles did not interfere with bone-healing processes after sinus augmentation procedures and promoted new bone formation. This study can help clinicians to understand better the morphological characteristics of bone regeneration processes using Bio-Oss after 20 months and, most importantly, after a longer	. Under transmission electron microscopy, it was possible to characterize the bone-biomaterial interface; in the 20-month specimen an electron-dense layer was seen, whereas, almost no electron-dense lines were seen at the interface in the 7-year specimen.	Histological and histo-morpho-metrical analysis, TEM	human	Case Report	Sinus Augmentation	anorganic bovine bone + collagen membrane	-	6 months
Carinci et al., Arch Oral Biol. 2006 [18]	he data reported are, to our knowledge, the first genetic portrait of Bio-Oss effects. They can be relevant to our improved understanding of the molecular mechanism underlying bone regenerative procedures and as a model for comparing other materials with similar clinical effects.	The log2 ratios for all the targets on the array were then calibrated using the normalization factor, and log2 ratios outside the 99.7% confidence interval (the median 3 times S.D. = 0.52) were determined as significantly changed in the treated cells.	Gene expression Microarray	osteoblast-like MG63 cells	In vitro study	Cell culture	anorganic bovine bone	Not treated cells	-
Orsini et al., J Biomed Mater Res B Appl Biomater. 2005 [14]	The analyses showed that Bio-Oss particles do not interfere with the normal osseous healing process after sinus lift procedures and promote new bone formation.	newly formed compact bone was present. In the first bone lamella collagen fibers contacting the Bio-Oss surface were oriented at 243.73 ± 7.12 degrees (mean ± SD), while in the rest of the lamella they were oriented at 288.05 ± 4.86 degrees (mean ± SD) with a statistically significant difference of 44.32 degrees (*p* < 0.001).	Histological and histo-morpho-metrical analysis, TEM, SEM	12 patients	Human	Sinus Augmentation	anorganic bovine bone	-	6 months
Mangano et al., J Oral Implantol. 2006 [43]	Intimate binding between bone and HA particles was present after a long-term implantation period (20 years). The fact that HA particles were surrounded closely by bone is very promising for the long-term stability of the augmentation.	Histomorphometry showed that bone represented 25.4 ± 3.2%, marrow spaces represented 41.3 ± 5.2%, and residual HA particles represented 38.1 ± 4.1%.	Histological and histo-morpho-metrical analysis	human	Case report	Post-extraction sockets	Dense hydroxyapatite	-	6 months

**Table 8:** Summary table of the advanced and custom-made experimental bone scaffold findings of the papers included.

Authors	Study Findings	Results	Biomaterials and Methodologies Characteristics	Study Model Model	Sample Size	Defect	Test Group	Control Group	Timepoints
[54]	-	-	-	-	-	-	-	-	-
Scarano et al., Biomed res int 2016 [1]	APG with β-TCP preserves skin morphology, without immune response, with an excellent tolerability and is a promising scaffold for cells and biomaterial for soft tissue augmentation. β-TCP added with APG was able to increase the bio-stimulating effect on fibroblasts and quicken resorption.	The margins of β-TCP granules were clear and not diffused near tissues.	The aim of the study was to evaluate microporous tricalcium phosphate (β-TCP) and autologous platelet gel (APG) mix in mice for oral and maxillofacial soft tissue augmentation.	in vivo Mice	10	Cheek	β-TCP/APG gel was injected into one cheek	β-TCP/APG gel was injected into one cheek; the other was used as control	-
[55]	-	-	-	-	-	-	-	-	-
Doi et al., PLoS ONE. 2012 [45]	IPCHA/implant complex might be able to achieve both bone reconstruction and implant stability. implant/interconnected porous hydroxyapatite complex as new concept graft material.	The ISQs of complex groups was 77.8 ± 2.9 in the 6-month, 72.0 ± 5.7 in the 3-month and 47.4 ± 11.0 in the 2-month. The BICs of complex groups was 2.18 ± 3.77 in the 2-month, 44.03 ± 29.58 in the 3-month, and 51.23 ± 8.25 in the 6-month.	ISQ measurement, histology	dog femur	4 animals	jaws defects	implant/interconnected porous hydroxyapatite complex	implants were placed directly into the femur without any bone substrate.	2, 6 months
Corinaldesi et al., Br J Oral Maxillofac Surg. 2013 [20]	In this pilot controlled trial of the use of rhBMP-7, histological analyses showed that it resulted in the formation of less bone than treatment with inorganic bovine hydroxyapatite.	Histological and histo-morpho-metric analyses of biopsy specimens showed that there was significantly more new bone on the control side (19.9 (6.8)%) than on the test side (6.6 (4.8)%).	Histological histo-morphometry	Human	9 patients/18 sinuses	Maxillary sinus	rhBMP-7 (Osigraft) with deproteinized bone substitute (0.5 g on the test side)	deproteinized bone alone (2.0 g on the control side)	6 months
Mangano et al., J Oral Implantol. 2010 [56]	Data from this case report demonstrate that the newly formed bone provided by engineered bone tissue	Augmented maxillary sinus with engineered bone presented a mean of 28.89% and 71.11% of bone and medullary spaces, respectively.	Histological histo-morphometry	Human	Case report	Maxillary sinus	autologous osteoblasts on polymeric scaffolds	-	6 months
Strocchi et al., J Oral Implantol. 2002 [57]	The presence of more blood vessels in the sites treated with CS could help to explain the good results reported in the literature with the use of CS.	The defects in group 3 (3 rabbits) were filled with autologous bone. A total of 54 defects were filled (18 with CS and e-PTFE membranes, 18 with CS alone, and 18 with autologous bone). No postoperative deaths or complications occurred. All nine animals were sacrificed at 4 weeks. MVD results were as follows: in the first group, 9.88 ± 4.613; in the second group, 7.92 ± 1.998; and in the third group, 5.56 ± 1.895. *p* = 0.000 was highly significant.	Histological histo-morphometry	rabbits	9 animals	tibial metaphysis	he defects were filled in a random way. The defects of group 1 (3 rabbits) were filled with CS granules (Surgiplaster, Classimplant, Rome, Italy) and covered with e-PTFE membranes. The defects in group 2 (3 rabbits) were filled with CS granules (Surgiplaster). The defects in group 3 (3 rabbits) were filled	autologous bone.	4 weeks
Scarano et al., Implant Dent. 2007 [58]	The results confirm the high biocompatibility and rapid resorption of calcium sulfate.	In light microscopy, trabecular bone was present. No remnants of calcium sulfate were present. Transmission electron microscopy showed, in the areas of the interface with the implant surface, features of mature bone with many osteocytes.	Histological histo-morphometry, SEM	Human	Case report	Peri-implant defect	Calcium sulfate	-	6 months
Serino et al., Clin Oral Implants Res. 2003. [59]	Alveolar bone resorption following tooth extraction may be prevented or reduced by the use of a bioabsorbable synthetic sponge of polylactide-polyglycolide acid. The quality of bone formed seemed to be optimal for dental implant insertion.	the mesial-buccal site, a loss of bone height of 0.2 mm (1.4 SD) in the test and 0.6 mm (1.1 SD) in the controls; in the mid-buccal portion a gain of 1.3 mm (1.9 SD) in the test and a loss of 0.8 mm (1.6 SD) in the controls; and in the distal portion a loss of 0.1 mm (1.1 SD) in the test and of 0.8 (1.5 SD) mm in the controls.	Histological histomorphometry,	Human	36 subjects	polylactide and polyglycolide sponge	Empty defect	-	6 months
Imbronito et al., J Biomed Mater Res A. 2005 [60]	In areas where the degrading copolymer formed accumulates, an amorphous multilayered material was identified between the connective tissue and the copolymer. In summary, the copolymer of PLA/PGA studied appears to be an osteoconductive material when it is used to fill bone defects.	In areas where the degrading copolymer was present in small amounts, newly formed bone matrix was detected; it was deposited by osteoblast-like cells in close relation to the copolymer	Histological histo-morphometry,	5 Rabbits	36 subjects	Maxillary sinus	polylactide and polyglycolide sponge	Empty defect	60 days
Carinci et al., J Craniofac Surg. 2006 [61]	he data reported are, to our knowledge, the first genetic portrait of osteoblast-like cells cultured on PP. They are relevant to better understanding of the molecular mechanism of bone-PP interaction and as a model for comparing other materials used for bone reconstruction.	(1) signal transduction, (2) transcription, (3) translation, (4) cell cycle regulation, (5) vesicular transport, and (6) production of cytoskeletal elements, cell-adhesion molecules and extracellular matrix components.	DNA microarrays	In vitro culture	osteoblast-like cells	osteoblast-like cell lines (i.e., MG-63)	Porous polyethylene	-	-

## Data Availability

All experimental data to support the findings of this study are available contacting the corresponding author upon request.
